# Injectable Amorphous Chitin-Agarose Composite Hydrogels for Biomedical Applications

**DOI:** 10.3390/jfb6030849

**Published:** 2015-08-25

**Authors:** Murali Vishnu Priya, Rajendran Arun Kumar, Amirthalingam Sivashanmugam, Shantikumar Vasudevan Nair, Rangasamy Jayakumar

**Affiliations:** Amrita Centre for Nanosciences and Molecular Medicine, Amrita Institute of Medical Sciences and Research Centre, Kochi 682041, India; E-Mails: mvishnupriya.2790@gmail.com (M.V.P.); drarunnano@gmail.com (R.A.K.); sivasa19619@aims.amrita.edu (A.S.); shantinair@aims.amrita.edu (S.V.N.)

**Keywords:** injectable hydrogel, amorphous chitin, agarose, biomedical applications

## Abstract

Injectable hydrogels are gaining popularity as tissue engineering constructs because of their ease of handling and minimal invasive delivery. Making hydrogels from natural polymers helps to overcome biocompatibility issues. Here, we have developed an Amorphous Chitin (ACh)-Agarose (Agr) composite hydrogel using a simpletechnique. Rheological studies, such as viscoelastic behavior (elastic modulus, viscous modulus, yield stress, and consistency), inversion test, and injectability test, were carried out for different ACh-Agr concentrations. The composite gel, having a concentration of 1.5% ACh and 0.25% Agr, showed good elastic modulus (17.3 kPa), yield stress (3.8 kPa), no flow under gravity, injectability, and temperature stability within the physiological range. Based on these studies, the optimum concentration for injectability was found to be 1.5% ACh and 0.25% Agr. This optimized concentration was used for further studies and characterized using FT-IR and SEM. FT-IR studies confirmed the presence of ACh and Agr in the composite gel. SEM results showed that the lyophilized composite gel had good porosity and mesh like networks. The cytocompatibility of the composite gel was studied using human mesenchymal stem cells (hMSCs). The composite gels showed good cell viability.These results indicated that this injectable composite gel can be used for biomedical applications.

## 1. Introduction

Hydrogels derived from biopolymers are gaining more interest in the field of tissue engineering because of their good biocompatibility and biodegradability. These gels, having a 3-D network, are very similar to the structure of extracellular matrix (ECM) of native human tissue/organs [1]. This highly networked structure provides good mechanical support, as well as good porosity, for cells to grow and proliferate. Good porosity also enables the efficient diffusion of solutes, including nutrients, and effusion of waste by-products [2]. Since our body is made up of 70% water, any material that can retain high amounts of water is ideal to be used as a tissue-engineering construct. Hydrogels can absorb as much as 1000 times its weight of water without undergoing dissolution [3].

Injectable hydrogels can be easily placed into the defect site with minimum invasiveness. Injectability helps in the homogeneous distribution of gel in the entire defect site [4]. This reduces the need for open surgery, thus, decreasing the chances of infection, affording a quicker healing time, and having a better aesthetic outcome. Injectable hydrogels have better adaptability to defect margins, moldability [5], handling properties, and cost effectiveness [6]. Different types of drugs, growth factors, biomolecules, and cells can be incorporated into hydrogels to enhance tissue regeneration [7,8].

Chitin is a naturally occurring biocompatible polysaccharide, containing *N-*acetyl-D-glucosamine units linked by β-1, 4-glycosidic bonds. It is suitable for tissue engineering applications as its monomeric units of *N*-acetyl glucosamine are similar to the composition of the ECM [9]. Because of this similarity, it can be easily degraded by the lysozymes present in the body [9].Chitin has been processed into scaffolds, sponges, membranes, microparticles, nanofibers, and nanogels, as well as hydrogels, for various tissue engineering and other biomedical applications [10,11,12,13,14]. However, the major drawback of chitin is the difficulty in dissolving it. The strong hydrogen bonding in the chitin polymer due to the high degree of acetylation (DAc) makes chitin insoluble in water [12]. Therefore, reducing the DAc increases the number of amino groups and breaks the secondary structure of chitin, thus making it amorphous (DAc ~ 70%–60%). This increases the hydrophilicity of chitin and makes it soluble in milder solvents [11,15,16] making it more cytocompatible. Usually, chitin is converted into chitosan, having a DAc less than 50%. This requires major chemical modifications. Thus, decreasing the DAc to around 60% would increase the solubility of chitin without using any major chemical modifications [17].

Agarose (Agr) is also a naturally occurring biocompatible linear polysaccharide, consisting of 1,4-linked 3,6-anhydro-α-L-galactose and 1,3-linked-β-D-*galactose* derivatives. Agr easily dissolves in water upon moderate heating and forms a gel at room temperature. However, Agr has a slow degradation rate [18], poor injectability, high hydrophilicity, and lower cell adhesiveness [19].To overcome these issues, Agr is blended with other polymers. Thus, a blend of ACh and Agr is expected to serve as a good injectable hydrogel for tissue engineering.

In this work, we propose a simple technique for the development of a composite hydrogel made of amorphous chitin (ACh) and agarose, which is injectable, and can be potentially used for the regeneration of soft tissues. Earlier similar works have been done using Chitosan and Agr [20,21,22]. However, these gels did not show injectable properties, which make their handling difficult for clinical applications. ACh gel, being injectable, is easy to handle and can fill in irregular defect sites with ease. Moreover, ACh is easy to process from chitin than chitosan, as chitosan requires extensive chemical modifications. Further, the monomers of ACh resemble the native glycosaminoglycan composition. Agr gel with its high water retaining capability mimics the water rich native ECM. Various concentrations of ACh and Agr solutions were used for making the hydrogels. The strength and injectability of the gels were analyzed, followed by *in vitro* studies to analyze the cytocompatibility of the optimized gel.

## 2. Results and Discussion

### 2.1. Preparation of ACh-Agr Composite Gel

**Figure 1:**
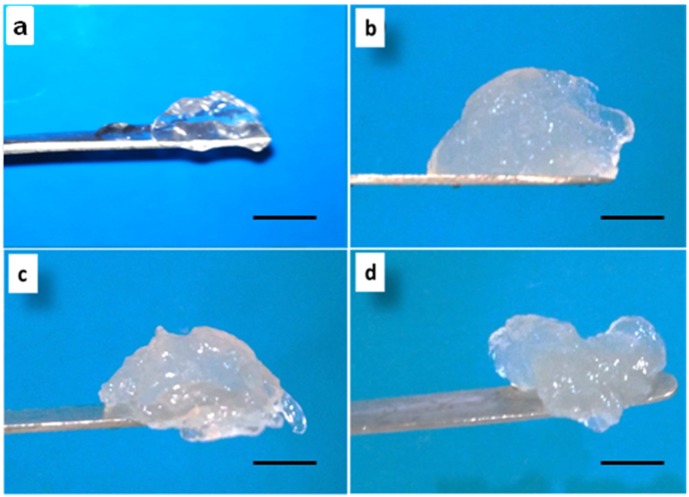
Photographic Images of (**a**) Agr gel; (**b**) ACh gel; (**c**) 1.5% ACh-0.25% Agr gel; and (**d**) 1.5% ACh-0.5% Agr gel. (Scale bar: 1 cm).

For preparing the Agr gel, Agr powder was dissolved in double distilled water and the solution was heated. Heated Agr solution, on cooling to room temperature, formed a transparent gel (Figure 1a). When heated, Agr molecules form double helical fibers having random coiled structures. These fibers, on cooling, aggregate into super coiled structures, thereby forming a gel [23].

ACh gel was formed by altering the pH. On adding excess of NaOH to ACh solution, translucent mass was observed which indicated the gel formation (Figure 1b). When ACh is dissolved in acidic pH, the polymeric chains break apart. On bringing the pH to alkaline, the chains move closer and get entangled [24]. Since the process takes place in an aqueous environment, water gets entrapped in between the polymer chains, thereby forming a hydrogel.

The ACh-Agr composite gel was prepared by simple regeneration method with help of heat treatment and pH change without any cross-linker. Upon altering the pH (to alkaline) of the heated ACh-Agr solution, the polymeric chains are expected to get entangled with each other and form a three-dimensional network, entrapping large amount of water (Figure 1c,d). The pH of the ACh and ACh-Agr gel was around 10, which was neutralized by subsequent washing with double distilled water.

### 2.2. Characterization of ACh-Agr Gel

**Figure 2:**
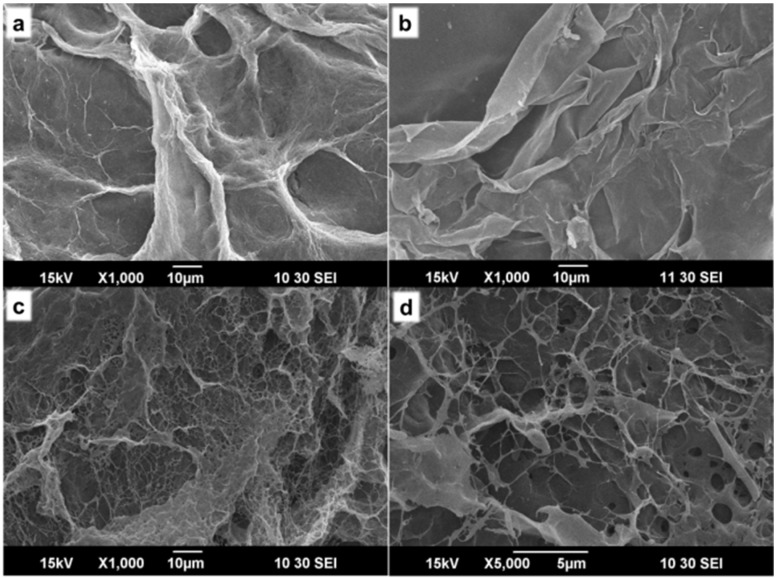
SEM images of (**a**) Agr (×1000); (**b**) ACh (×1000); (**c**) ACh-Agr (×1000) and (**d**) ACh-Agr (×5000).

The lyophilized composite and control gels were analyzed using SEM. It was observed that the Agr gel showed a slight fiber like morphology, forming a mesh like network (Figure 2a). The ACh gel showed no porosity (Figure 2b) and had smooth morphology. The ACh-Agr gel showed mesh like network with interconnected micro-porous structure (Figure 2c,d). This porous structure can enhance the efficient transport of solute molecules when placed at the tissue site and help the cells to grow and proliferate within the gel.

**Figure 3:**
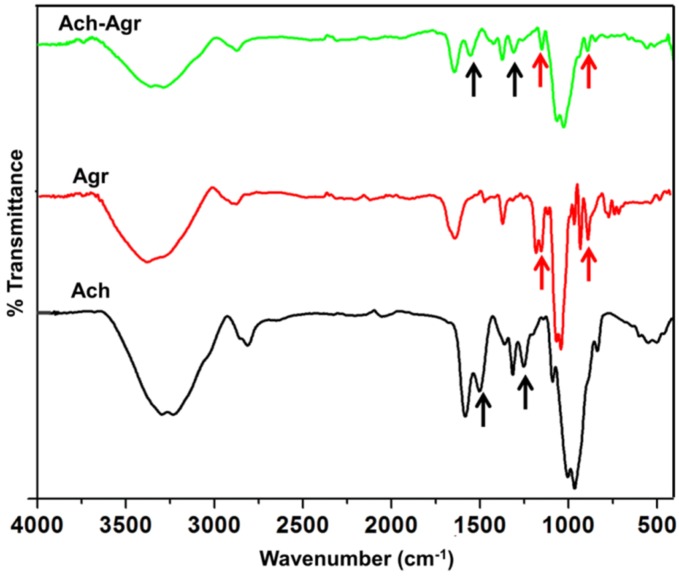
FTIR spectrum of ACh, Agr and ACh-Agr hydrogel.

Further the composite gel was characterized using FTIR spectroscopy (Figure 3). The FT-IR spectra of ACh showed peaks at 1660, 1500, 1455, 1020 and 951 cm^−1^ [17]. Agr showed its characteristic peaks at 1046, 1371, 890 and 931 cm^−1^ [20]. In ACh-Agr gel, the Agr peaks at 1046 and 931 cm^−1^were seen with slight change, which may be because of its interaction with ACh. 1046 cm^−1^peak represents the C–O axial deformation and 931 cm^−1^ represents 3,6-anhydrogalactose group in Agr. ACh peak at 1500 and 1250 cm^−1^were retained in the composite ACh-Agr gel. The 1500 cm^−1^ peak represents-C stretching of the glycosidic ring and 1250 cm^−1^ representsthe C–N stretching. Therefore FT-IR confirmed the presence of ACh and Agr in the composite gel.

### 2.3. Rheological Analysis of Composite Gels

**Figure 4:**
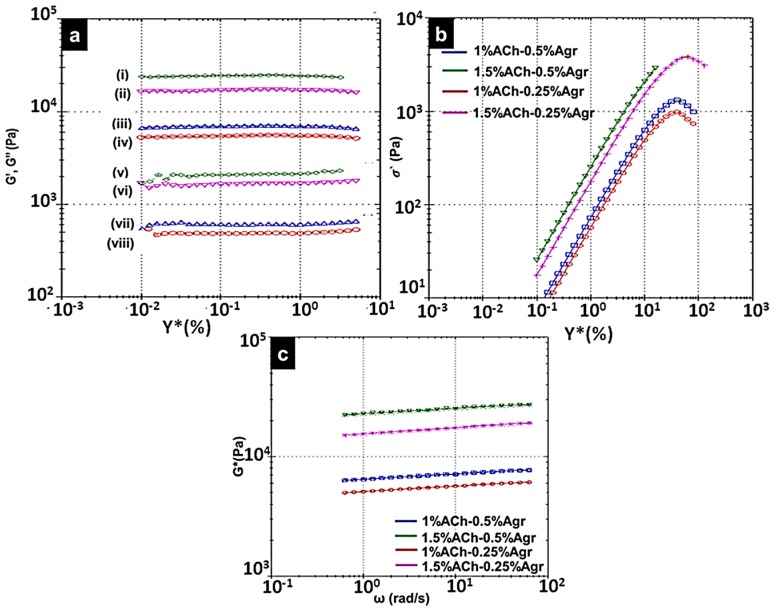
Rheological properties of the composite gels (**a**) Amplitude sweep, showing the elastic modulus (i,iv) and viscous modulus(v–viii) at different shear strain. (i,v) 1.5% ACh-0.5% Agr; (ii,vi) 1.5% ACh-0.25% Agr; (iii,vii) 1% ACh-0.5% Agr; (iv,viii) 1% Ach-0.25% Agr). (**b**) Yield stress analysis. (**c**) Power law model fit data for different compositions of the hydrogels.

Various concentrations of ACh and Agr solutions were used to make hydrogels in different compositions: (i) 1% ACh-0.25% Agr (ii) 1% ACh-0.5% Agr (iii) 1.5% ACh-0.25% Agr and (iv) 1.5% ACh-0.5% Agr. All the four samples were subjected to rheological analysis (Figure 4). To study the physical nature of the gel, amplitude sweep was performed (Figure 4a). Amplitude sweep gives the elastic modulus (*G*′) and viscous modulus (*G*′′) for different shear strain (%). Shear strain (0.01%–100%) was applied to determine the Linear Viscoelastic Region (LVER), the region in which the sample can elastically strain and return to its original state once the strain is removed. All further rheological analysis was performed in this LVER. Elastic modulus represents the solid component and viscous modulus represents the liquid component of the material under testing. For a gelling material, the solid component must be more than the liquid component. It was noticed that all the compositions showed higher elastic modulus than the viscous modulus, thus confirming the gel nature of the composite. 1% ACh-0.25% Agr gel had an elastic modulus of 5.6 kPa. This value increased to 7.1 kPa, when the Agr concentration was increased to 0.5%. Similar results were obtained for gels made of 1.5% ACh. The elastic modulus of 1.5% ACh-0.25% Agr gel was found to be 17.3 kPa and for 1.5% ACh-0.5% Agr it was 25 kPa. The gels made of 1.5% ACh showed higher elastic modulus as compared to gels made of 1% ACh. The gel with the composition of 1.5% ACh and 0.5% Agr showed highest elastic modulus (*G*′) (Table 1).

Figure 4b shows the yield stress analysis of the gels. The composite gel containing 1.5% ACh and 0.25% Agr had a higher yield stress (sigma prime) at 3.8 kPa, as compared to the other hydrogels, which implies that this particular composition of hydrogel is able to take higher loads. Interestingly, though the composition having 1.5% ACh and 0.5% Agr showed higher elastic modulus (high *G*′ value), it could not withstand higher loads and thus started to yield at around 2.9 kPa. It was observed that on increasing the concentration of ACh, the yield stress value increased, however an increase in the Agr concentration showed different trends when added to 1% and 1.5% ACh. The gel containing 1% ACh and 0.25% Agr started to yield at 0.98 kPa. This value increased to 1.3 kPa for 1% ACh-0.5% Agr gel. In the case of 1.5% ACh gels, the gel having 0.5% Agr started to yield at 2.9 kPa, whereas the gel with 0.25% Agr had a yield stress of 3.8 kPa. This may be because, on increasing the concentration of ACh, the gel might be becoming more viscous and with addition of higher amounts of Agr, the gel may start to become slightly brittle and hence lose its load bearing ability. Based on the yield stress analysis, 1.5% ACh-0.25% Agr gel was found to be better than 1.5% ACh-0.5% Agr gel.

Power law model fit data was generated by the rheometer (Figure 4c). This data along with the power law equation (Equation (1)) was used to obtain the power law index (*n*), consistency (*k*) and the correlation coefficient (Corr. Coeff) values (Table 1). The *n* value is a measure of the shear thinning property of the gel. Shear thinning is the property of the gel, which makes it flow only under shear (external force). Without an external shear the gel should behave as a solid. This *n* value is important when considering the injectability of the gel. For a shear thinning gel *n* value is expected to be below 1(for water *n* = 1, pure solid *n* = 0) [25]. Based on the power law analysis it was found that all the compositions of hydrogels had a shear thinning property with *n*<1. The k value helps us to understand the consistency of the gel. Higher the *k* value, higher the viscosity of the gel. Gels made of 1% ACh-0.25% Agr and 1% ACh-0.5% Agr had a *k* value of 5.11 kPa and 6.48 kPa, respectively. On the other hand, 1.5% ACh-0.25% Agr and 1.5% ACh-0.5% Agr showed a *k* value of 15.5 kPa and 22.9 kPa, respectively. It was observed that on increasing the Agr concentration, the gel became more viscous. This result was consistent with the low yield stress value of the 1.5% ACh-0.5% Agr gel. The Corr. Coeff gives the closeness of the original data to the model fit data. All the gel composition had a Corr. Coeff > 0.99 (perfect correlation = 1), thereby showing that the gels followed the model fit values (Figure 4c).

σ = *kΫ^n^* (1)

where, σ = Shear stress; *k* = Consistency; *Ϋ* = Shear rate; *n* = Power law index.

**Table 1:** Rheological properties for different concentrations of composite ACh-Agr gels.

Amorphous Chitin (%)	Agarose (%)	*G*′ (kPa)	Sigma Prime (Pa)	Complex Shear Strain (%)	*n*	*k* (kPa)	Corr. Coeff
1	0.25	5.6	0.981 × 10^3^	40.27	0.04488	5.11	0.9985
1	0.5	7.1	1.3 × 10^3^	42.33	0.04433	6.48	0.9983
1.5	0.25	17.3	3.8 × 10^3^	62.54	0.05155	15.5	0.9981
1.5	0.5	25	2.9 × 10^3^	15.88	0.04389	22.9	0.9917

**Figure 5:**
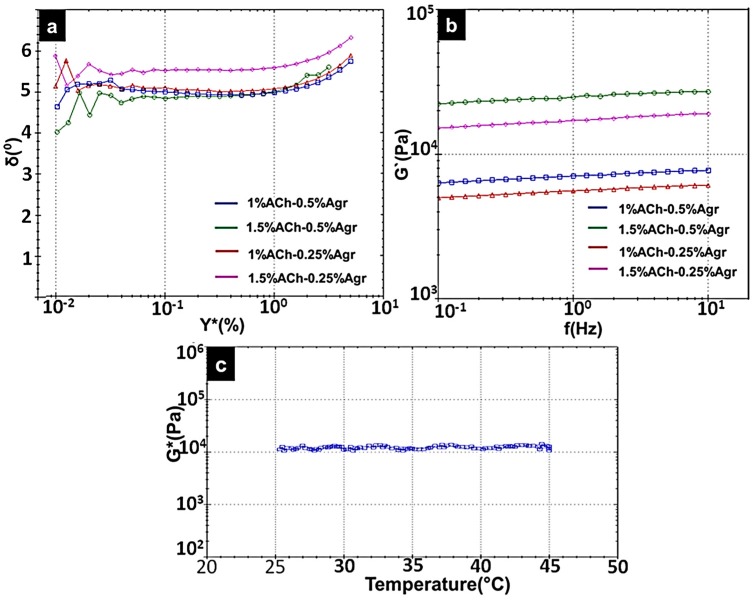
(**a**) Phase angle *vs.* shear strain; (**b**) Frequency sweep (Elastic Modulus *vs.* Frequency) for different composite ACh-Agr hydrogels; (**c**) Temperature stability of 1.5% ACh-0.25% Agr hydrogel.

The phase angle values for all the gels were between 4° and 6° (Figure 5a).This indicates that the gels are of solid dominating nature with a liquid component (δ = 0° for purely solid, and δ = 90°, for purely liquid) [26].

Frequency sweep determines the elastic modulus with respect to time (inversely proportional to frequency) (Figure 5b). It was observed that as the frequency decreased (increase in time), the *G*′ values did not show any considerable amount of variation. A very slight decrease in the elastic modulus was observed, which may be due to the water content.

Since 1.5% ACh-0.25% Agr gels showed good yield stress along with higher elastic modulus and shear thinning property, further, its temperature stability was tested between 25 °C and 45 °C. It was observed that the complex modulus (G*) remained more or less constant (Figure 5c). This shows that the gel is able to withstand considerable amount of temperature variability.

**Figure 6:**
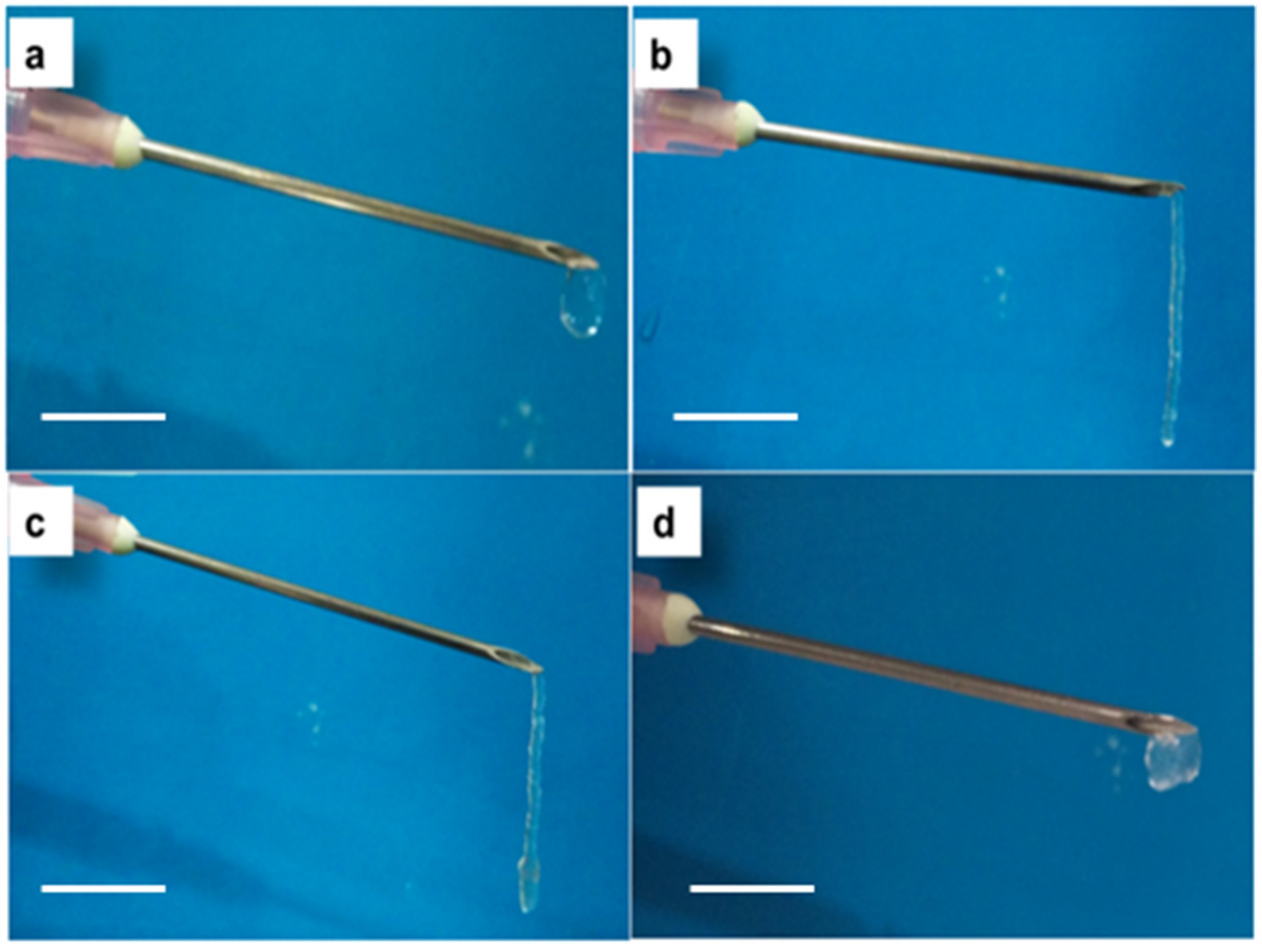
Injectability test: (**a**) 0.25% Agr; (**b**) 1.5% ACh; (**c**) 1.5% ACh-0.25% Agr; (**d**) 1.5% ACh-0.5% Agr. (Scale bar: 1 cm).

As all the gels showed *n* < 1, they were all expected to be injectable. In order to confirm this, injectability test was done. Gels having 1.5% ACh were used for these tests as they showed higher modulus and consistency. Injectability test was carried out using 18 G needle by applying shear manually. The injectability of the gels was compared with the control gels, and it was observed that 0.25% Agr gel (Figure 6a) was dripping when shear was applied where as 1.5% ACh showed considerable amount of injectability but with breaks in the flow (Figure 6b). The gel made of 1.5% ACh-0.25% Agr showed good and continuous injectability (Figure 6c).This injectable property of the 1.5% ACh-0.25% Agr gel could enable one to deliver it at the damaged tissue site with minimum invasiveness. Gel with a composition of 1.5% ACh-0.5% Agr did not show injectability (Figure 6d). This may be due higher Agr concentration. Since 1.5% ACh-0.25% Agr gel showed good viscoelastic behavior, along with good injectability, this composition was chosen for further studies.

**Figure 7:**
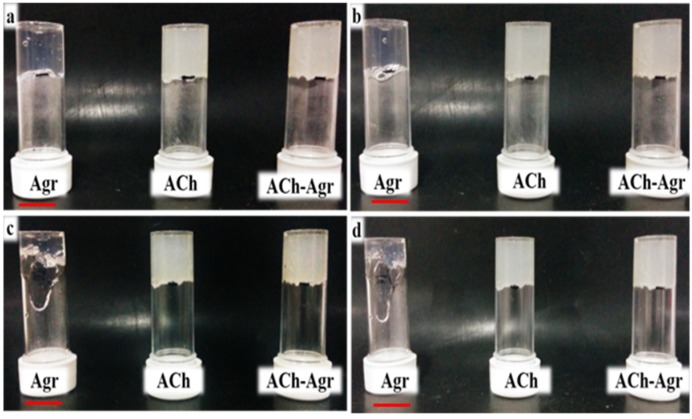
Inversion test for Agr, ACh and 1.5% ACh-0.25% Agr at different time points (**a**) 0 h; (**b**) 0.5 h; (**c**) 1 h; (**d**) 24 h. (Scale bar: 1 cm).

Inversion test was carried out for 1.5% ACh gel, 0.25% Agr gel and 1.5% ACh-0.25% Agr gel. This test is used to observe the effect of gravity on the gel flow. The flow of the hydrogels was visually observed at different time points. Agr gel started to flow within half an hour of inversion (Figure 7b), whereas the other two gels showed no flow even after 24 h of inversion (Figure 7c,d). The early flow of the Agr gel may be due to the low adhesiveness of the Agr molecules. The adhesive forces between the Agr molecules are expected to be too low to overcome the shear by gravitational force. ACh gel showed no flow even after 24 h of inversion. This could be attributed to higher adhesive forces between the ACh molecules. Thus, the ACh-Agr hydrogel is expected to stay in the defect site and not flow off to the neighboring tissue till the damaged tissue is repaired.

### 2.4. Cytocompatibility

**Figure 8:**
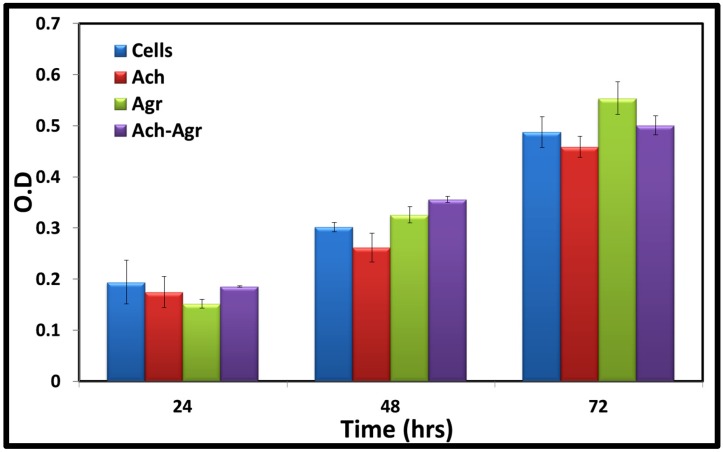
hMSCs viability was analyzed on ACh, Agr and ACh-Agr hydrogels at different time points (24, 48 and 72 h) using AlamarBlue^®^ assay.

Cytocompatibility of the composite gel was studied using human Mesenchymal Stem Cells(hMSCs) by AlamarBlue^®^ (Thermo Fisher Scientific, Waltham, MA, USA) assay for 24, 48 and 72 h (Figure 8). All the gels were cytocompatible up to 72 h. There was no significant difference between the cell viability of the 1.5% ACh gel, 0.25% Agr gel and composite gels (1.5% ACh-0.25% Agr). With increase in time, there was an increase in cell number, indicating the proliferation of the hMSCs. Control group (cells alone) and the gel groups showed similar amount of cell viability, thereby indicating the non-toxic nature of the gels. As these gels were prepared under mild conditions, the cytocompatibility of the polymers was not affected by the preparation technique.

## 3. Experimental Section

### 3.1. Materials

ACh (DAc 60%) was purchased from Koyo Chemicals Ltd. (Tokyo, Japan). Agr (Ultrapure), Iscove’s Modified Dulbecco’s Medium (IMDM), Mesenchymal Stem Cells (MSCs) specific Foetal Bovine Serum (FBS), Penicillin, Streptomycin and AlamarBlue^®^ dye were bought from Invitrogen (Camarillo, CA, USA). Acetic acid was obtained from Sigma Aldrich (Bangalore, India). Sodium hydroxide (NaOH) was purchased from Qualigens (Qualigens Fine Chemicals, Mumbai, India). Double distilled (MQ) water (18.2 MΩ·cm) was used wherever needed. All other chemicals used were of analytical grade.

### 3.2. Preparation of ACh-Agr Composite Gel

**Figure 9:**
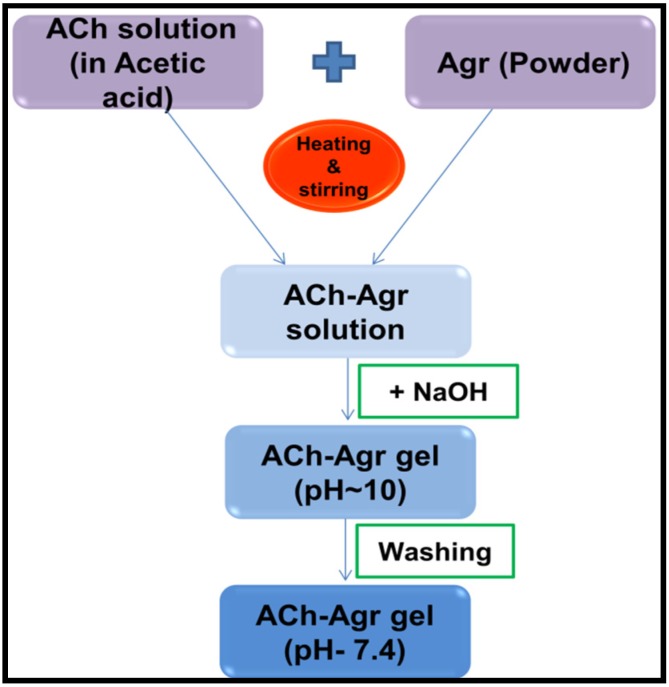
Schematic of preparation of composite ACh-Agr hydrogel.

Different concentrations of ACh (1% and 1.5%) and Agr solutions (0.25% and 0.5%) were made and mixed in varying proportions (Table 1). ACh solutions were made by dissolving the required concentration in 1% acetic acid. 1N NaOH was slowly added to ACh solution to form ACh gel. The ACh gel was formed at pH 10. Agr was dissolved in MQ water with mild heating to form the Agr solution, which formed a gel on cooling. In order to make ACh-Agr gel, the Agr powder was added to the ACh solution under constant stirring and mild heating. On complete dissolution of the Agr in the ACh solution, 1N NaOH was added under constant stirring and cooled to room temperature. Four samples were made, 1% ACh with 0.25% and 0.5% Agr and 1.5% ACh with 0.25% and 0.5% Agr. In order to remove the excess NaOH, the gels are washed several times (Figure 9). Pure ACh and Agr gels were used as controls.

### 3.3. Physicochemical Characterization of the Gel

The control and composite gels were lyophilized for 24 h. The porosity and morphology of the gels were characterized using Scanning Electron Microscope (JEOL JSM-6490LA Analytical SEM). In order to compare the functional groups in the control and composite gels, Fourier Transform Infra-Red (FTIR) Spectroscopy (Shimadzu IRAffinity-1S Fourier Transform Infrared Spectrophotometer) was carried out.

### 3.4. Rheological Studies

#### 3.4.1. Viscoelastic Studies

In order to assess the strength, flow and stability of the gels, Rheological tests were carried out using a Malvern Kinexus pro rheometer. Amplitude sweep, frequency sweep, yield stress analysis and power law model fit analysis were performed using a stainless steel 20 mm cone plate (4°) (upper). The gap between upper and lower plates was kept at 0.5 mm.The instrument was set to determine the LVER and frequency sweep was carried out. Elastic modulus (*G*′), viscous modulus (*G*′′) and phase angle (δ) were measured against varying percentages of shear strain. The yield stress analysis was done between 10 Hz to 0.1 Hz to find out the yielding point of the gel. All the experiments were carried out at 25 °C.

#### 3.4.2. Temperature Stability

In order to determine the temperature stability of the composite gel, it was subjected to varying temperatures from 25 to 45 °C and oscillatory tests were carried out. Throughout the experiment, a constant frequency and shear was maintained and the complex modulus and complex viscosity were measured.

#### 3.4.3. Injectability andInversion Study

The injectability of the gels was tested by loading them into a 1 mL syringe (with 18 G needle) and subjecting them to manual shear. The flow was observed visually. Inversion test was done to analyze the effect of gravity on the gels’ flow/stability. 0.25% Agr, 1.5% ACh and the composite gels (1.5% ACh-0.25% Agr) were subjected to this test. Equal volumes of these three gels were placed in a cylindrical vial (with flat bases) and their levels were labeled. The vials were inverted and made to stand on their caps and were left undisturbed [27]. The flow of the gels was observed at different time intervals (0 h, 0.5 h, 1 h and 24 h).

### 3.5. Cytocompatibility

AlamarBlue^®^ assay was carried out to determine the cell viability and proliferation. In this assay the blue, non-fluorescent resazurin dye (AlamarBlue^®^ reagent) is reduced to a pink, fluorescent resorufin compound by the metabolically active enzymes in the live cells. Adipose derived hMSCs were used to carry out the cell viability studies. Equal amounts of UV-sterilized composite and control hydrogels were taken in a 12 well plate and hMSCs were seeded at a density of 15,000 cells/well. IMDM supplemented with 10% FBS and streptomycin/penicillin was used as the growth medium. The cells were then incubated at 37 °C for different time periods of 24, 48 and 72 h. After incubating for the required time periods, the culture media was removed and 10% AlamarBlue^®^ reagent in IMDM was added to each of the wells and incubated at 37 °C. After 5 h incubation, the optical density is measured at 570 nm with 600 nm as the reference wavelength using a microplate spectrophotometer (Biotek Power Wave XS, Winooski, VT, USA) [28].

### 3.6. Statistical Analysis

All experiments were carried out in triplicates. Statistical significance was analyzed using two-tailed paired Student’s t-test. *p* < 0.05 was considered significant.

## 4. Conclusions

The ACh-Agr composite hydrogels were prepared under mild conditions and characterized. Viscolelastic behavior of the gels was studied to know their injectable nature. It was observed that 1.5% ACh-0.25% Agr gels showed good viscoelastic behavior along with good injectability. Inversion test showed that this gel flowed only under shear, thereby withstanding flow under gravity. Further, cytocompatibility of the composite gel was proved using hMSCs. These studies demonstrated that gels with the composition of 1.5% ACh and 0.25% Agr can be used for biomedical applications. Furthermore, this gel system would serve as a base for further modifications and incorporation of fillers, growth factors, biomolecules, *etc.*, to improve the functionalities.
